# Different survival analysis methods for measuring long-term outcomes of Indigenous and non-Indigenous Australian cancer patients in the presence and absence of competing risks

**DOI:** 10.1186/s12963-016-0118-9

**Published:** 2017-01-17

**Authors:** Vincent Y. F. He, John R. Condon, Peter D. Baade, Xiaohua Zhang, Yuejen Zhao

**Affiliations:** 1 Menzies School of Health Research, Charles Darwin University, PO Box 41096, Casuarina, NT 0811 Australia; 2 Cancer Council Queensland, PO Box 201, Spring Hill, QLD 4004 Australia; 3 Northern Territory Government Department of Health, Health Gains Planning Branch, PO Box 40596, Casuarina, NT 0811 Australia

**Keywords:** Survival analysis, Indigenous Australians, Competing risks, Fine-Gray model, Net survival, Crude probability of death, Cancer prognosis, Life tables, Smoking, Cause of death data

## Abstract

**Background:**

Net survival is the most common measure of cancer prognosis and has been used to study differentials in cancer survival between ethnic or racial population subgroups. However, net survival ignores competing risks of deaths and so provides incomplete prognostic information for cancer patients, and when comparing survival between populations with different all-cause mortality. Another prognosis measure, “crude probability of death”, which takes competing risk of death into account, overcomes this limitation. Similar to net survival, it can be calculated using either life tables (using Cronin-Feuer method) or cause of death data (using Fine-Gray method). The aim of this study is two-fold: (1) to compare the multivariable results produced by different survival analysis methods; and (2) to compare the Cronin-Feuer with the Fine-Gray methods, in estimating the cancer and non-cancer death probability of both Indigenous and non-Indigenous cancer patients and the Indigenous cancer disparities.

**Methods:**

Cancer survival was investigated for 9,595 people (18.5% Indigenous) diagnosed with cancer in the Northern Territory of Australia between 1991 and 2009. The Cox proportional hazard model along with Poisson and Fine-Gray regression were used in the multivariable analysis. The crude probabilities of cancer and non-cancer methods were estimated in two ways: first, using cause of death data with the Fine-Gray method, and second, using life tables with the Cronin-Feuer method.

**Results:**

Multivariable regression using the relative survival, cause-specific survival, and competing risk analysis produced similar results. In the presence of competing risks, the Cronin-Feuer method produced similar results to Fine-Gray in the estimation of cancer death probability (higher Indigenous cancer death probabilities for all cancers) and non-cancer death probabilities (higher Indigenous non-cancer death probabilities for all cancers except lung cancer and head and neck cancers). Cronin-Feuer estimated much lower non-cancer death probabilities than Fine-Gray for non-Indigenous patients with head and neck cancers and lung cancers (both smoking-related cancers).

**Conclusion:**

Despite the limitations of the Cronin-Feuer method, it is a reasonable alternative to the Fine-Gray method for assessing the Indigenous survival differential in the presence of competing risks when valid and reliable subgroup-specific life tables are available and cause of death data are unavailable or unreliable.

**Electronic supplementary material:**

The online version of this article (doi:10.1186/s12963-016-0118-9) contains supplementary material, which is available to authorized users.

## Background

In Australia, Indigenous cancer patients have lower net survival than non-Indigenous patients [1], but there is limited information about whether Indigenous cancer patients also have higher probability of non-cancer death, given the Indigenous population’s higher prevalence of chronic diseases and much higher all-cause mortality rates [2].

Net survival is the measure most commonly used to compare cancer prognosis in different populations (e.g., between nations, regions, or racial/ethnic groups) [1, 3–9] because net survival removes the effect of non-cancer deaths (which may vary between populations because of different all-cause mortality rates [10]) and measures the hypothetical scenario in which patients are only able to die of their cancer. However, this is a disadvantage of net survival for patients and clinicians who, when considering the treatment options and weighing up the benefits, drawbacks, and toxicities of cancer therapy, want to know about actual prognosis: what is the chance of dying (from cancer or any cause) compared with the chance of surviving [5, 9, 10]? This is particularly important for Indigenous Australians, and other populations who also have higher probability of death from “competing” (i.e., non-cancer) causes [3, 5, 10].

For this purpose, “crude probability of death” is a more appropriate measure of prognosis than net survival because it takes competing risk into account and enables us to identify whether survival disparities are caused by cancer deaths, non-cancer deaths, or both. Crude probability of cancer death (hereafter referred to as “cancer death probability”) is the probability of death from cancer in the presence of other causes; while crude probability of non-cancer death (hereafter referred to as “non-cancer death probability”) is the probability of death from other causes in the presence of cancer.

Both net survival and crude probability of death can be estimated in two different ways: one based on cause of death to classify each death as a cancer or non-cancer death, the other based on life tables to compare the number of deaths in cancer patients with the number “expected” based in total population mortality rates (Table 1). Cause of death data can be used to estimate net survival by calculating the cause-specific survival proportion or to estimate crude probabilities of cancer and non-cancer death using the Fine-Gray competing risks model. Similarly, life tables can be used to calculate net survival using the relative survival method or crude probabilities of cancer and non-cancer deaths using the Cronin-Feuer method.

**Table 1:** A summary of different cancer prognosis statistics and survival analysis methods used in this study

Prognosis statistics		Using cause of death data	Using life tables
Net survival (excludes competing risks)	Measure	Cause-specific survival	Relative survival
	Calculation method	Kaplan-Mier	Divide observed survival by expected survival
	Multivariable analysis method	Cox proportional hazard model	Poisson model
Crude probability (includes competing risks)	Measure	Crude probability of death	Crude probability of death using life table
	Calculation method	Fine-Gray method	Cronin-Feuer method
	Multivariable analysis method	Fine-Gray model	Nil

Life table-based methods are commonly used for comparing cancer survival between population groups because of deficiencies in cause of death data; such deficiencies may be greater for disadvantaged subgroups. However, life tables are often not available for such subgroups because of uncertainty about population estimates and absent or incomplete identification of subgroup members in death registrations. Australia has high-quality cause of death data but incomplete life tables for the Indigenous population, except for the Northern Territory (NT) where Indigenous Australians are very high prevalence (30% of the NT population compared to 2–4% in other states) and consequently are accurately identified in deaths data and have reliable population estimates [11]. Indigenous status is also recorded with a high degree of accuracy in the NT Cancer Register (NTCR) [12]. The availability of high-quality data for the Indigenous population and Indigenous cancer patients puts the NT in the best position to compare the implications of using the Cronin-Feuer and Fine-Gray methods to assess the Indigenous disparities in cancer and non-cancer deaths.

The aim of this study is two-fold: (1) to compare the multivariable results (i.e., Indigenous differentials) produced by relative survival, cause-specific survival, and competing risk (Fine-Gray) survival analysis methods; and (2) to compare the Cronin-Feuer with the Fine-Gray survival analysis methods, in estimating the cancer and non-cancer death probability of both Indigenous and non-Indigenous cancer patients and the Indigenous cancer disparities in the presence of competing causes of death.

## Methods

### Data

Cancer registrations data for all NT residents diagnosed with cancer between 1 January 1991 and 31 December 2009 was obtained from the NTCR for the following data items: sex; date of birth; Indigenous status; remoteness of residence (urban or remote); date of diagnosis; cancer site, coded according to the International Classification of Diseases 10 (ICD-10); date of death; and underlying cause of death. Vital status was verified by matching the NTCR dataset to the National Death Index for deaths occurring up to 31 December 2011 (at the time of analysis, cause of death data were only available up until 31 December 2011). For the survival analysis of site-specific cancers, female breast (C50), colorectal (C18-20), lung (C33-34), and head and neck (C1-C14 & C30-32) cancers were chosen because there were sufficient numbers of both Indigenous and non-Indigenous cases for the analysis, and they are Australia’s designated national “priority” cancers [13] (except head and neck cancers). Head and neck cancer was chosen as it is a major cause of morbidity and mortality associated with smoking.

### Statistical analysis

The censoring date for the survival analysis was 31 December 2011. The survival time for cancer patients who died on the day of diagnosis was counted as half a day.

Relative survival was calculated using the “strs” procedures [14] of Stata, in which the expected survival was estimated using the Ederer II method [15]. The NT non-Indigenous population has very high migration to and from other parts of Australia and similar health status and mortality to Australians generally, so life tables for the relevant years (1991–2009) for the total Australian population were used for non-Indigenous cancer patients. Life tables for the NT Indigenous population (for which deaths and population data are more reliable than for Indigenous people in other states and territories [11]) were used for the Indigenous cancer patients. Cancer and non-cancer death probabilities were calculated in two ways: the Fine-Gray model used cause of death information from the cancer cohort, and was derived using the “stcrreg” command in Stata, while the Cronin-Feuer method used life table data [16] derived using the “strs” command in Stata [14].

For multivariable analysis, the Cox proportional hazard regression, Poisson regression (generalized linear model with Poisson error structure), and Fine-Gray regression were used to investigate the Indigenous survival disparity; multivariable analysis was not available for the Cronin-Feuer method. The same independent variables were included in all final models: Indigenous status (Indigenous compared to non-Indigenous); age at diagnosis (per year); gender (female compared with male); and cancer site. Since the effect of age at diagnosis was found to be different for Indigenous compared with non-Indigenous patients, an interaction term for Indigenous status by age at diagnosis (base age 55 years) was added to the models. For the Cox proportional hazard regression, scaled Schoenfeld residuals were used to check if the proportional hazards assumptions of each variable were satisfied. As the proportional hazards assumption was not met for Indigenous status, a step function of Indigenous status with follow-up time (as annual intervals) was also included in the model.

In the multivariable analysis for time trends, as all subjects had at least 2 years of potential follow-up, the follow-up was limited to the first 2 years after diagnosis so that the shorter follow-up time for subjects diagnosed late in the study period did not bias time trends. Regression models included the same terms as above, plus year of diagnosis.

All analyses were conducted using Stata/SE 13 (StataCorp, College Station, TX). Ethics approval was obtained from the Human Research Ethics Committee of the Menzies School of Health Research and NT Department of Health. Approval to use the cancer registrations data was obtained from the NT Cancer Registry.

## Results

There were 9,595 invasive cancer cases between 1991 and 2009 that fulfilled the study criteria (Table 2). 18.6% were identified as Indigenous, who were more likely to be female, younger, and live in remote regions. A higher proportion of Indigenous (69.8%) than non-Indigenous (46.4%) patients died during the study period.

**Table 2:** Demographic characteristics of people diagnosed with cancer, Australia NT, 1991–2009

	Indigenous (*n* = 1789)	Non-Indigenous (*n* = 7806)
Sex (%)		
Male	46.5	59.1
Female	53.5	40.9
Age at diagnosis (%)		
0 to 49 years	36.3	30.3
50 to 59 years	26.6	26.0
60 to 69 years	22.0	23.7
70 years and over	15.1	20.0
Median age (years)	55	57
Remoteness (%)		
Urban	29.9	84.8
Remote	70.1	15.2
Vital Status at 31 Dec 2011 (%)		
Alive	30.2	53.6
Dead	69.8	46.4

Adjusting for age and sex, net survival (estimated using relative survival) was lower for Indigenous than non-Indigenous patients at both 1 and 5 years after diagnosis for breast, colorectal, and head and neck cancers (Table 3).

**Table 3:** One-year and 5-year cumulative relative survival (%) and 95% confidence interval by Indigenous status and cancer site (age and sex adjusted), Australia NT, 2001–2009

	Indigenous			Non-Indigenous		
Cancer site	Cases	One-year	Five-year	Cases	One-year	Five-year
Breast	152	87.3 (80.1–92.1)	66.4 (55.7–75.1)	954	97.7 (95.9–98.7)	86.5 (83.1–89.3)
Colorectal	96	64.3 (53.6–73.1)	40.4 (29.1–51.4)	784	84.2 (81.3–86.8)	58.4 (54.2–62.4)
Head and neck^a^	194	54.0 (46.5–60.9)	36.0 (28.5–43.6)	514	85.7 (82.3–88.5)	63.9 (59.2–68.2)
Lung^a^	293	27.6 (22.5–33.0)	8.9 (5.7–12.9)	740	36.0 (32.1–39.8)	12.6 (9.9–15.6)

^a^Smoking-related cancers

The multivariable results for all cancers combined (adjusted for cancer site) were similar for all terms included in the model for each of the three methods: relative survival analysis using Poisson regression; cause-specific analysis using Cox proportional hazards regression; and competing risk analysis using Fine-Gray regression (Tables 4 and 5). The Poisson regression, Cox proportional hazards regression, and the Fine-Gray regression (for cancer deaths) showed that the excess mortality of Indigenous patients was highest in the first year after diagnosis and decreased over time until the fifth year, with male and older patients experiencing higher risk of cancer death (Table 4). Age at diagnosis had different effects for Indigenous and non-Indigenous cases; the cancer death rate increased by 1% per year of age for Indigenous cases and 3% per year of age for non-Indigenous cases (Table 4). The Fine-Gray model (for non-cancer deaths) showed that Indigenous patients were more likely than non-Indigenous to die of non-cancer causes and that, in contrast to cancer deaths, this differential increased rather than decreased with time in the first 5 years after diagnosis (Table 4, Additional file 1: Table S3a). Remoteness of residence at time of diagnosis was not associated with risk of either cancer or non-cancer death for Indigenous or non-Indigenous patients and was therefore not included in all the regression models. The results for the analysis of time trend in 2-year survival rate were similar for all three regression models (Table 5, Additional file 2: Table S4a); the death rate in the first 2 years after diagnosis decreased by 3% per diagnosis year after adjustment for Indigenous status, sex, and age.

**Table 4:** Regression analysis of cause-specific mortality (Cox proportional hazard regression), relative survival (Poisson regression), and competing risk (Fine-Gray regression), all cancers combined^a^, Australia NT, 1991–2009

	Relative survival	Cause-specific	Competing (due to cancers)	Competing (other death)
	HR (95% CI)^b^	HR (95% CI)	SHR (95% CI)	SHR (95% CI)
Indigenous^c^				
1st year after diagnosis	2.15 (1.96–2.36)	2.17 (1.98–2.38)	1.97 (1.79–2.17)	1.67 (1.16–2.42)
2nd year after diagnosis	1.35 (1.11–1.64)	1.47 (1.22–1.76)	1.53 (1.27–1.85)	2.75 (1.57–4.81)
3rd year after diagnosis	1.09 (0.79–1.52)	1.32 (1.01–1.72)	1.45 (1.11–1.90)	5.10 (2.89–8.99)
4th year after diagnosis	1.29 (0.83–1.99)	1.26 (0.87–1.80)	1.44 (1.00–2.08)	8.96 (4.60–17.47)
5th year after diagnosis	0.56 (0.25–1.28)	0.78 (0.46–1.32)	0.95 (0.56–1.62)	5.72 (2.75–11.88)
Female vs male	0.83 (0.77–0.90)	0.84 (0.78–0.90)	0.85 (0.78–0.92)	0.71 (0.54–0.93)
Age at diagnosis^d^				
Non-Indigenous	1.03 (1.03–1.03)	1.03 (1.03–1.03)	1.03 (1.02–1.03)	1.07 (1.06–1.08)
Indigenous	1.02 (1.01–1.02)	1.01 (1.01–1.02)	1.01 (1.00–1.02)	1.05 (1.03–1.07)

^a^Model adjusted for cancer site (with colorectal cancer as the reference category for cancer site)

^b^ *HR* hazard ratio, *SHR* standard hazard ratio

^c^Applies to the reference categories of the interaction terms (i.e., people of median age 55 years)

^d^Per year of age

**Table 5:** Regression analysis of time trend after diagnosis using cause-specific mortality (Cox proportional hazard regression), relative survival (Poisson regression), and competing risk analysis (Fine-Gray regression), all cancers combined^a^, Australia NT, 1991–2009

	Relative survival	Cause-specific	Competing (due to cancers)	Competing (other death)
	HR (95% CI)^b^	HR (95% CI)	SHR (95% CI)	SHR (95% CI)
Indigenous^c^	1.99 (1.82–2.16)	2.01 (1.85–2.19)	1.94 (1.77–2.13)	2.49 (1.70–3.63)
Female vs male	0.86 (0.79–0.93)	0.84 (0.77–0.91)	0.87 (0.79–0.94)	0.74 (0.53–1.04)
Age at diagnosis^d^				
Non-Indigenous	1.03 (1.03–1.04)	1.03 (1.03–1.04)	1.03 (1.03–1.03)	1.07 (1.06–1.08)
Indigenous	1.02 (1.01–1.02)	1.02 (1.01–1.02)	1.02 (1.01–1.02)	1.02 (1.00–1.04)
Year of diagnosis	0.97 (0.96–0.97)	0.97 (0.96–0.97)	0.97 (0.96–0.97)	0.97 (0.95–1.00)

^a^Model adjusted for cancer site (with colorectal cancer as the reference category for cancer site)

^b^ *HR* hazard ratio, *SHR* standard hazard ratio

^c^Applies to the reference categories of the interaction terms (i.e. people of median age 55 years in 2009)

^d^Per year of age

The measures of crude probabilities of deaths are presented as proportion of cases that have died at 5 years after diagnosis (Table 6) from cancer and other causes. Cancer death probabilities (for all cancers combined and for the four individual cancer sites) were higher for Indigenous than non-Indigenous patients 5 years after cancer diagnosis, whether calculated by the Fine-Gray or Cronin-Feuer methods. Non-cancer death probabilities were also higher for Indigenous than non-Indigenous patients for all cancers combined and for breast and colorectal cancers, as estimated by both the Cronin-Feuer and Fine-Gray methods. Amongst the patients with head and neck cancers and lung cancer (both smoking-related cancers) and colorectal cancer, the non-cancer death probabilities estimated by Cronin-Feuer were much lower than those estimated by the Fine-Gray methods for the non-Indigenous, but not the Indigenous patients. Amongst the patients with breast cancer, the cancer and non-cancer death probabilities estimated by Cronin-Feuer were higher than those estimated by the Fine-Gray methods. The Indigenous disparities (in cancer death) estimated by the Cronin-Feuer method were lower than those estimated by the Fine-Gray method for all cancers except for breast cancer; the Indigenous disparities (in non-cancer death) estimated by the Cronin-Feuer method were higher than those estimated by the Fine-Gray method for all cancers except for lung cancer and head and neck cancers.

**Table 6:** Five-year cumulative probabilities of cancer and non-cancer death (%), age and sex adjusted

	Cancer type	Indigenous			Non-Indigenous			Disparity	
		Cronin-Feuer	Fine-Gray	Ratio (CF/FG)	Cronin-Feuer	Fine-Gray	Ratio (CF/FG)	CF^a^	FG^b^
Cancer death probabilities	All cancers	61.0	65.7	0.93	33.2	33.2	1.00	1.84	1.98
	Breast	31.6	28.9	1.09	13.2	12.2	1.08	2.39	2.37
	Colorectal	57.7	65.1	0.89	40.6	40.1	1.01	1.42	1.62
	Head & neck^c^	62.5	67.7	0.92	35.8	33.6	1.07	1.75	2.01
	Lung^c^	88.4	92.5	0.96	86.4	85.5	1.01	1.02	1.08
Other causes	All cancers	6.1	4.0	1.53	3.7	3.0	1.23	1.65	1.33
	Breast	10.6	4.3	2.47	4.4	2.0	2.20	2.41	2.15
	Colorectal	7.6	6.6	1.15	4.4	5.3	0.83	1.73	1.25
	Head & neck^c^	6.2	5.9	1.05	2.8	8.2	0.34	2.21	0.72
	Lung^c^	4.0	3.0	1.33	1.6	3.9	0.41	2.50	0.77

^a^Disparity measured using Cronin-Feuer method (expressed as ratio of death probabilities among the Indigenous cancer cohort to the death probabilities among the non-Indigenous cohort diagnosed with the same cancer)

^b^Disparity measured using Fine-Gray method

^c^Smoking-related cancers

## Discussion

This study is the first to examine the implications of various survival methods to assess the survival inequalities faced by Indigenous cancer patients in the NT, in particular the impact of competing causes of death. Multivariable regression using relative survival (Poisson model), cause-specific survival (Cox regression) and competing risk analysis (Fine-Gray model) produced similar results. For all cancers combined, death rates were higher for Indigenous, male and older cancer patients for both cancer and non-cancer death. In the absence of competing risk, Indigenous people have lower 5-year net survival than non-Indigenous people for all three cancers (breast, colorectal, and head and neck cancers).

Crude probability of death potentially offers a more understandable way than net survival to communicate cancer prognosis to patients and clinicians, showing the different mortality risk profiles (cancer and non-cancer death probabilities) among the Indigenous and non-Indigenous populations, while acknowledging that cancer patients can also die of non-cancer causes [17]. It enables us to identify whether survival disparities are caused by cancer, non-cancer, or both cancer and non-cancer deaths, as well as identify the disparities in both cancer and non-cancer deaths across time. The multivariable competing risk analysis, Fine-Gray model, found that Indigenous patients were more likely than non-Indigenous to die of non-cancer causes and that, in contrast to cancer deaths, this differential increased rather than decreased with time after diagnosis. Five years after diagnosis, Indigenous cancer patients had higher rates of non-cancer death than non-Indigenous patients and this differential was increasing with time since diagnosis, while there was no longer a differential in cancer deaths. This finding suggests the need for integrated chronic disease management strategies for cancer patients, particularly for the Indigenous population with higher rate of chronic diseases comorbidities. The finding also demonstrates an important advantage of the competing risk approach over conventional relative survival in analyzing long-term outcomes for Indigenous cancer patients.

In the presence of competing risks, the Cronin-Feuer method produced similar results to the Fine-Gray method in the estimation of cancer death probability (higher Indigenous cancer death probabilities for all cancers) and non-cancer death probabilities (higher Indigenous non-cancer death probabilities for all cancers except lung cancer and head and neck cancers) 5 years after cancer diagnosis. The Cronin-Feuer method estimated much lower non-cancer death probabilities than the Fine-Gray method for non-Indigenous patients with head and neck cancers and lung cancer, which are both smoking-related cancers.

Comparison of death probabilities estimated using the Cronin-Feuer method and Fine-Gray models provides insights into “external factors” affecting measures of survival for various cancer types such as smoking-related cancers. The death probabilities estimated using the Cronin-Feuer method can be seen as the death probabilities that the cancer patients were “expected to have”, in the hypothetical scenario where cancer patients have the same death risks as the general population, unaffected by “external factors” that cancer patients experienced such as unhealthy behaviors, access to health care, or screening effects. The death probabilities estimated using cause of death data and the Fine-Gray method can be seen as the “observed death probabilities” in which cancer patients might have different risks of death to the general population due to various “external factors”. Therefore, higher (or lower) non-cancer death probabilities estimated by the Fine-Gray model imply higher (or lower) non-cancer death risks in cancer patients than the general population.

When analyzing smoking-related cancers, caution is required for prognosis measures calculated using general population life tables such as relative survival and the Cronin-Feuer method [18–20], which assume that cancer patients have the same death risks as the general population. This assumption is probably violated for smoking-related cancers, because smoking increases mortality from other causes of death such as respiratory and cardiovascular diseases, resulting in higher non-cancer death risks in cancer patients than the general population. Therefore, the Cronin-Feuer method will underestimate the non-cancer death probabilities of the patients with smoking-related cancers if it uses the general population life tables for the cancer patients with higher non-cancer death risks. The results from our study support the hypothesis that methods using life tables (relative survival and Cronin-Feuer) underestimate non-cancer deaths and overestimate cancer deaths for smoking-related cancers for non-Indigenous patients, suggesting that Cronin-Feuer method should be used with caution in estimating cancer and non-cancer death probability for non-Indigenous patients with smoking related cancers and quantifying the Indigenous disparity for smoking-related cancers. Our study has shown that the Indigenous disparity in smoking-related cancer death probabilities estimated by Cronin-Feuer method is lower than that estimated by the Fine-Gray method, which suggests that survival analysis methods that use the life table such as relative survival might underestimate the Indigenous disparity in cancer survival for smoking-related cancers.

Previous studies suggested that relative survival tends to underestimate social inequalities in cancer survival [21–23]. Our study suggests that the Cronin-Feuer method does underestimate Indigenous to non-Indigenous disparities in cancer death probabilities (except for breast cancer) while overestimating disparities in non-cancer death probabilities (except for lung, head and neck cancers, which are both smoking-related cancers) after considering competing risks. Underestimation of cancer death disparity by the Cronin-Feuer method was largest for colorectal cancer and head and neck cancers. For colorectal cancer, this underestimation was mainly due to underestimation of the probability of cancer death for Indigenous patients. For head and neck cancers, underestimation of disparity is due to a combination of underestimation of probability of cancer death for Indigenous patients and overestimation of cancer death probabilities for non-Indigenous patients. However, for breast cancer patients the disparity in cancer deaths was similar for the two methods; the Fine-Gray method produced lower estimates of both cancer and non-cancer death probability than the Cronin-Feuer method for both Indigenous and non-Indigenous patients.

The lower estimates of cancer death probabilities of the Fine-Gray method might indicate that cancer survival for breast cancer patients is better than expected (calculated using life table). The lower estimates of non-cancer death probabilities of the Fine-Gray method might indicate lower non-cancer 5-year death risk in breast cancer patients than the background population, supported by the findings of another study [24] (not specifically for Indigenous cancer patients) that breast cancer patients have reduced risks for death (compared to the general population) from cardiovascular disease (SMR = 83.9), which accounts for the majority (55%) of non-cancer deaths among cancer patients generally in Australia; higher risks for non-cancer deaths were observed for all cancer types except breast cancer and melanoma. The lower than expected cancer and non-cancer death probability for breast cancer patients may be due to these patients having higher socioeconomic status, which might be a protective factor of cancer and non-cancer deaths, as indicated by the finding that breast cancer is more common in areas of higher socioeconomic status in Australia [25] (breast cancer incidence rate was 122 per 100,000 in the highest socioeconomic status group, compared to 103 per 100,000 in the lowest socioeconomic status group).

## Conclusion

While net survival is useful for reporting trends in cancer survival, comparing different groups of cancer patients and investigating the impact of various factors on cancer treatment, crude probability of death is important when communicating risks to patients [10] during clinical decision making [9, 26]. Previous studies have suggested that relative survival tends to underestimate social inequalities in cancer survival; our study suggests that the Cronin-Feuer method does underestimate Indigenous to non-Indigenous disparities in cancer death probabilities (except for breast cancer) in the presence of competing risks. Our study also suggests that when analyzing smoking-related cancers, caution is required when measuring cancer survival disparity using population life tables. Despite the limitations of the Cronin-Feuer method [16], it is a reasonable alternative to the Fine-Gray method for assessing the Indigenous survival differential in the presence of competing risks when valid and reliable subgroup-specific life tables are available and cause of death data are unavailable or unreliable.

## Additional files

Additional file 1: Table S3a. Regression analysis of cause-specific mortality (Cox proportional hazard regression), relative survival (Poisson regression), and competing risk (Fine-Gray regression), all cancers combined^1^, Australia NT, 1991–2009 (full model). Description: **Table S3a.** including hazard ratios for specific cancer sites. (DOC 49 kb)
Additional file 2: Table S4a. Regression analysis of time trend after diagnosis using cause-specific mortality (Cox proportional hazard regression), relative survival (Poisson regression), and competing risk analysis (Fine-Gray regression), all cancers combined^1^, Australia NT, 1991–2009 (full model). Description: **Table S4a.** including hazard ratios for specific cancer sites. (DOC 47 kb)
