# Supplementary material for: Different survival analysis methods for measuring long-term outcomes of Indigenous and non-Indigenous Australian cancer patients in the presence and absence of competing risks
Source: Popul Health Metr. 2017 Jan 17;15:1. doi: 10.1186/s12963-016-0118-9 (PMC5240232; doi:10.1186/s12963-016-0118-9)
Supplement: Additional file 1: Table S3a. — Regression analysis of cause-specific mortality (Cox proportional hazard regression), relative survival (Poisson regression), and competing risk (Fine-Gray regression), all cancers combined1, Australia NT, 1991–2009 (full model). Description: Table S3a. including hazard ratios for specific cancer sites. (DOC 49 kb) [file 12963_2016_118_MOESM1_ESM.doc]

Additional file 1: Table S3a: Regression analysis of cause-specific mortality (Cox proportional hazard regression), relative survival (Poisson regression), and competing risk (Fine-Gray regression), all cancers combined^1^, Australia NT, 1991-2009 (full model). Description: Table S3a including hazard ratios for specific cancer sites.

|  | **Relative survival** | **Cause-specific** | **Competing (due to cancers)** | **Competing (other death)** |
| --- | --- | --- | --- | --- |
|  | **HR (95% CI)^2^** | **HR (95% CI)** | **SHR (95% CI)** | **SHR (95% CI)** |
| Indigenous**^3^** |  |  |  |  |
| 1st year after diagnosis | 2.15 (1.96-2.36) | 2.17 (1.98-2.38) | 1.97 (1.79-2.17) | 1.67 (1.16-2.42) |
| 2nd year after diagnosis | 1.35 (1.11-1.64) | 1.47 (1.22-1.76) | 1.53 (1.27-1.85) | 2.75 (1.57-4.81) |
| 3rd year after diagnosis | 1.09 (0.79-1.52) | 1.32 (1.01-1.72) | 1.45 (1.11-1.90) | 5.10 (2.89-8.99) |
| 4th year after diagnosis | 1.29 (0.83-1.99) | 1.26 (0.87-1.80) | 1.44 (1.00-2.08) | 8.96 (4.60-17.47) |
| 5th year after diagnosis | 0.56 (0.25-1.28) | 0.78 (0.46-1.32) | 0.95 (0.56-1.62) | 5.72 (2.75-11.88) |
| Female vs male | 0.83 (0.77-0.90) | 0.84 (0.78-0.90) | 0.85 (0.78-0.92) | 0.71 (0.54-0.93) |
| Age at diagnosis**^4^** |  |  |  |  |
| Non-Indigenous | 1.03 (1.03-1.03) | 1.03 (1.03-1.03) | 1.03 (1.02-1.03) | 1.07 (1.06-1.08) |
| Indigenous | 1.02 (1.01-1.02) | 1.01 (1.01-1.02) | 1.01 (1.00-1.02) | 1.05 (1.03-1.07) |
| Cancer sites^5^ |  |  |  |  |
| Head and neck | 1.17 (1.00-1.37) | 1.14 (0.98-1.32) | 1.14 (0.98-1.32) | 1.05 (0.66-1.65) |
| Stomach | 2.97 (2.49-3.54) | 2.84 (2.39-3.36) | 2.79 (2.37-3.28) | 0.56 (0.26-1.19) |
| Liver | 3.81 (3.06-4.74) | 3.34 (2.69-4.16) | 2.95 (2.28-3.82) | 1.14 (0.52-2.48) |
| Pancreas | 6.73 (5.52-8.21) | 5.94 (4.88-7.23) | 5.74 (4.65-7.09) | 0.35 (0.11-1.14) |
| Lung | 3.94 (3.47-4.48) | 3.68 (3.26-4.16) | 3.56 (3.15-4.03) | 0.45 (0.28-0.73) |
| Bone | 0.97 (0.67-1.41) | 0.93 (0.65-1.34) | 0.91 (0.62-1.31) | 1.26 (0.43-3.70) |
| Skin cancers | 0.18 (0.14-0.23) | 0.22 (0.18-0.27) | 0.22 (0.18-0.27) | 0.73 (0.45-1.18) |
| Breast | 0.32 (0.26-0.40) | 0.38 (0.31-0.46) | 0.38 (0.32-0.46) | 0.89 (0.54-1.45) |
| Female genital cancers | 0.98 (0.80-1.19) | 0.96 (0.80-1.16) | 0.94 (0.78-1.14) | 1.29 (0.75-2.22) |
| Male genital cancers | 0.23 (0.18-0.30) | 0.30 (0.25-0.36) | 0.31 (0.26-0.37) | 0.99 (0.67-1.46) |
| Kidney | 0.78 (0.59-1.04) | 0.72 (0.55-0.96) | 0.71 (0.54-0.94) | 1.57 (0.84-2.92) |
| Bladder | 0.58 (0.44-0.76) | 0.57 (0.45-0.74) | 0.59 (0.46-0.75) | 1.22 (0.73-2.04) |
| Brain | 3.31 (2.65-4.14) | 3.27 (2.63-4.06) | 3.22 (2.60-3.99) | 0.40 (0.10-1.65) |
| Thyroid | 0.30 (0.17-0.55) | 0.32 (0.19-0.53) | 0.32 (0.19-0.52) | 1.73 (0.80-3.75) |
| Lymphoma | 0.92 (0.74-1.15) | 0.93 (0.75-1.15) | 0.92 (0.75-1.13) | 0.85 (0.44-1.66) |
| Leukemia | 1.59 (1.27-1.99) | 1.48 (1.19-1.85) | 1.44 (1.14-1.83) | 1.43 (0.78-2.65) |
| Unknown primary | 4.44 (3.83-5.16) | 4.09 (3.54-4.73) | 3.92 (3.32-4.63) | 0.48 (0.25-0.91) |
| Others | 0.99 (0.82-1.19) | 0.87 (0.73-1.05) | 0.83 (0.69-1.00) | 1.82 (1.20-2.76) |

^1^Model adjusted for cancer site (with colorectal cancer as the reference category for cancer site).

^2^HR=hazard ratio; SHR=standard hazard ratio.

^3^Applies to the reference categories of the interaction terms (i.e., people of median age 55 years).

^4^ Per year of age.

^5^ Compared to colorectal cancer.
