# Supplementary material for: Different survival analysis methods for measuring long-term outcomes of Indigenous and non-Indigenous Australian cancer patients in the presence and absence of competing risks
Source: Popul Health Metr. 2017 Jan 17;15:1. doi: 10.1186/s12963-016-0118-9 (PMC5240232; doi:10.1186/s12963-016-0118-9)
Supplement: Additional file 2: Table S4a. — Regression analysis of time trend after diagnosis using cause-specific mortality (Cox proportional hazard regression), relative survival (Poisson regression), and competing risk analysis (Fine-Gray regression), all cancers combined1, Australia NT, 1991–2009 (full model). Description: Table S4a. including hazard ratios for specific cancer sites. (DOC 47 kb) [file 12963_2016_118_MOESM2_ESM.doc]

Additional file 2: Table S4a: Regression analysis of time trend after diagnosis using cause-specific mortality (Cox proportional hazard regression), relative survival (Poisson regression), and competing risk analysis (Fine-Gray regression), all cancers combined^1^, Australia NT, 1991-2009 (full model). Description: Table S4a including hazard ratios for specific cancer sites.

|  | **Relative Survival** | **Cause-specific** | **Competing (due to cancers)** | **Competing (other death)** |
| --- | --- | --- | --- | --- |
|  | **HR (95% CI)^2^** | **HR (95% CI)** | **SHR (95% CI)** | **SHR (95% CI)** |
| Indigenous**^3^** | 1.99 (1.82-2.16) | 2.01 (1.85-2.19) | 1.94 (1.77-2.13) | 2.49 (1.70-3.63) |
| Female vs male | 0.86 (0.79-0.93) | 0.84 (0.77-0.91) | 0.87 (0.79-0.94) | 0.74 (0.53-1.04) |
| Age at diagnosis**^4^** |  |  |  |  |
| Non-Indigenous | 1.03 (1.03-1.04) | 1.03 (1.03-1.04) | 1.03 (1.03-1.03) | 1.07 (1.06-1.08) |
| Indigenous | 1.02 (1.01-1.02) | 1.02 (1.01-1.02) | 1.02 (1.01-1.02) | 1.02 (1.00-1.04) |
| Year of diagnosis | 0.97 (0.96-0.97) | 0.97 (0.96-0.97) | 0.97 (0.96-0.97) | 0.97 (0.95-1.00) |
| Cancer sites^5^ |  |  |  |  |
| Head and neck | 1.31 (1.09-1.56) | 1.27 (1.07-1.51) | 1.28 (1.08-1.53) | 1.07 (0.58-1.98) |
| Stomach | 3.47 (2.86-4.21) | 3.06 (2.54-3.70) | 3.05 (2.54-3.68) | 0.74 (0.30-1.82) |
| Liver | 4.82 (3.81-6.09) | 4.00 (3.17-5.05) | 3.69 (2.83-4.82) | 1.43 (0.57-3.58) |
| Pancreas | 9.11 (7.39-11.24) | 6.86 (5.57-8.45) | 6.81 (5.52-8.39) | 0.59 (0.18-1.98) |
| Lung | 4.77 (4.13-5.52) | 4.05 (3.52-4.67) | 4.01 (3.47-4.62) | 0.68 (0.38-1.21) |
| Bone | 1.04 (0.68-1.61) | 0.94 (0.61-1.46) | 0.93 (0.60-1.44) | 2.48 (0.83-7.39) |
| Skin cancers | 0.19 (0.14-0.26) | 0.21 (0.16-0.28) | 0.22 (0.16-0.28) | 0.74 (0.38-1.43) |
| Breast | 0.24 (0.18-0.33) | 0.28 (0.21-0.36) | 0.28 (0.22-0.36) | 0.60 (0.28-1.30) |
| Female genital cancers | 1.04 (0.83-1.30) | 0.99 (0.79-1.23) | 0.98 (0.79-1.22) | 1.47 (0.73-2.98) |
| Male genital cancers | 0.25 (0.19-0.33) | 0.29 (0.23-0.36) | 0.29 (0.23-0.36) | 1.15 (0.68-1.94) |
| Kidney | 0.86 (0.62-1.20) | 0.80 (0.58-1.10) | 0.78 (0.56-1.09) | 1.78 (0.79-4.01) |
| Bladder | 0.70 (0.52-0.94) | 0.74 (0.56-0.97) | 0.75 (0.58-0.99) | 0.82 (0.35-1.90) |
| Brain | 4.19 (3.28-5.35) | 3.75 (2.95-4.77) | 3.76 (2.97-4.76) | 0.74 (0.18-3.08) |
| Thyroid | 0.29 (0.14-0.61) | 0.34 (0.18-0.62) | 0.34 (0.18-0.62) | 1.03 (0.24-4.42) |
| Lymphoma | 1.08 (0.84-1.39) | 1.05 (0.83-1.35) | 1.04 (0.81-1.32) | 1.17 (0.53-2.60) |
| Leukemia | 2.05 (1.60-2.61) | 1.91 (1.50-2.43) | 1.87 (1.45-2.42) | 1.62 (0.73-3.62) |
| Unknown primary | 5.70 (4.83-6.73) | 4.79 (4.08-5.64) | 4.68 (3.92-5.59) | 0.77 (0.37-1.58) |
| Others | 1.24 (1.01-1.52) | 1.12 (0.91-1.37) | 1.09 (0.88-1.34) | 2.09 (1.21-3.64) |

**^1^**Model adjusted for cancer site (with colorectal cancer as the reference category for cancer site).

**^2^**HR=hazard ratio; SHR=standard hazard ratio.

**^3^**Applies to the reference categories of the interaction terms (i.e., people of median age 55 years in 2009).

**^4^** Per year of age.

^5^ Compared to colorectal cancer.
